# A Comparision of Nalbuphine with Morphine for Analgesic Effects and Safety : Meta-Analysis of Randomized Controlled Trials

**DOI:** 10.1038/srep10927

**Published:** 2015-06-03

**Authors:** Zheng Zeng, Jianhua Lu, Chang Shu, Yuanli Chen, Tong Guo, Qing-ping Wu, Shang-long Yao, Ping Yin

**Affiliations:** 1 Medical Student, Department of Epidemiology and Biostatistics, School of Public Health, Tongji Medical College, Huazhong University of Science and Technology; 2 WuXiPRA Clinical Research (Shanghai) Co., Ltd, Shanghai, China; 3 Professor, Department of Anesthesiology, University Hospital of Xiehe, Wuhan, China

## Abstract

Although morphine is the standard opioid analgesic for pain control and has been widely used, certain drug-induced adverse effects have been reported as intolerable and need to be addressed. Nalbuphine may have a few advantages over morphine in this respect. We aimed to describe the effect of nalbuphine as well as its saftey compared to morphine by analyzing published randomized controlled trials (RCTs) with meta-analysis approach. We analysed 15 trials (820 patients). Overall, there was no evidence to show that the effect of pain relief had any difference between nalbuphine and morphine (pooled relative risks [RRs], 1.01; 95% CI, 0.91 to 1.11; *P* = 0.90). On the other hand, the incidences of pruritus, nausea, vomiting, respiratory depression were significantly lower in nalbuphine group compared with morphine group, and the pooled RRs were 0.78(95%CI, 0.602–0.997; *P* = 0.048) for nausea, 0.65(95%CI, 0.50–0.85; *P* = 0.001) for vomiting, 0.17(95%CI, 0.09–0.34; *P* *<* 0.0001) for pruritus, and 0.27(95%CI, 0.12–0.57; *P* = 0.0007) for respiratory depression. The analgesic efficacy of nalbuphine is comparable to morphine, but nalbuphine provides a better safety profile than morphine in the aspect of certain side-effects, especially related to pruritus and respiratory depression.

Opioids are commonly used as analgesics during the perioperative period, which is an integral part of the treatment of pain due to surgery and labour^1^. Morphine is the standard opioid analgesic for pain control. When it is used appropriately, about 80% of patients will achieve adequate pain relief^2^. However, many patients may change to an alternative opioid, because of the intolerable adverse effects associated with morphine.

Nalbuphine is an opioid agonist-antagonist of the phenanthrene series which was synthesized in an attempt to provide analgesia without the undesirable side effects of the pure agonists^3^. Its analgesic and possibly certain anti-pruritic effects are mediated via actions on the μ and κ-receptors, and nalbuphine has been indicated for mild to moderate pain^4^. It has been shown to be safe and effective when used for the treatment of conditions ranging from burns, multiple trauma, orthopaedic injuries, gynaecology and intra-abdominal conditions^5^,^6^.

However, the comparative results of efficacy and safety between morphine and nalbuphine are inconsistent among literatures. Therefore, there is no enough evidence to show which one is better in the treatment of pain due to surgery and labour. Perhaps nalbuphine may have advantages over morphine in the aspect of the adverse events. So we have conducted a meta-analysis of RCTs to determine the efficacy and safety of nalbuphine compared with morphine.

## Results of meta-analysis

### Search Results And Reporting Quality

We formulated a comprehensive search strategy to identify relevant studies regardless of language and publication status. Fifteen RCTs^3^,^7^,^8^,^9^,^10^,^11^,^12^,^13^,^14^,^15^,^16^,^17^,^18^,^19^,^20^ were included in our meta-analysis including 820 patients. First, we made use of the ten studies^7^,^8^,^9^,^10^,^11^,^12^,^13^,^14^,^15^,^16^ to evaluate analgesic efficacy camparing nalbuphine with morphine. The other four studies^3^,^17^,^18^,^19^ which also discussed about nalbuphine and morphine did not report the incidence of pain relief about nalbuphine and morphine, and the study by Etches *et al.*^20^ reported a small sample size (15 patients only). Second, a comparision of nalbuphine with morphine for clinical safety was also conducted. Six trials reported pruritus^10^,^12^,^15^,^16^,^17^,^20^, twelve studies reported nausea^3^,^7^,^8^,^9^,^10^,^12^,^14^,^15^,^16^,^17^,^18^,^19^, ten trials reported vomiting^3^,^8^,^9^,^10^,^11^,^12^,^13^,^14^,^15^,^19^, and three trials reported respiratory depression^11^,^17^,^20^. Thus, we have extracted information from those studies to evaluate the incidence of pruritus, nausea, vomiting and respiratory depression respectively between nalbuphine groups and morphine groups. Table 1 shows the details of retrieved studies^7^,^8^,^9^,^10^,^11^,^12^,^13^,^14^,^15^,^16^ about nalbuphine and morphine for analgesic effects.

### Pain Relief

**Figure 1:**
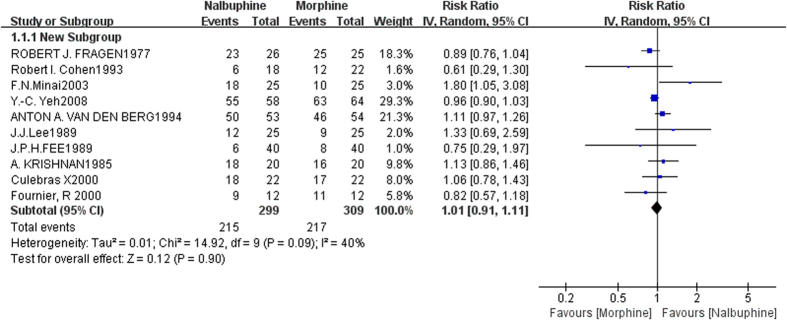
Forest plot of the incidence of pain relief comparing nalbuphine and morphine.

**Figure 2:**
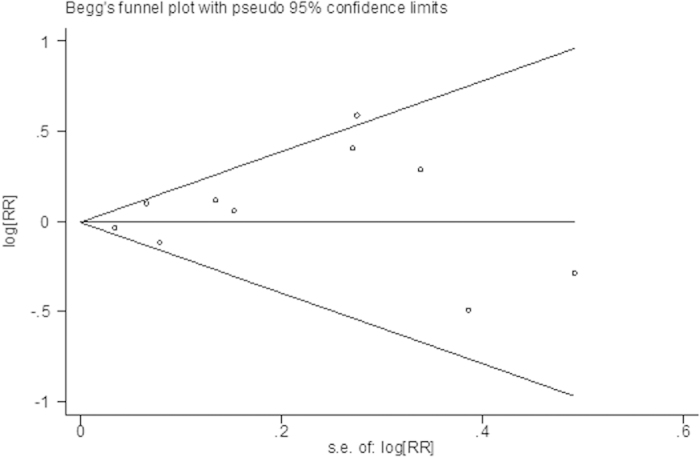
Funnel plot of the incidence of pain relief comparing nalbuphine and morphine.

Data was got from ten studies^7^,^8^,^9^,^10^,^11^,^12^,^13^,^14^,^15^,^16^, including 618 patients (nalbuphine/morphine: 299/309). In pooled analyses, there was no significant difference of incidence of pain relief between nalbuphine and morphine (the pooled RRs,1.01; 95% confidence interval [CI], 0.91 to 1.11; *P* = 0.90) (Fig. 1). There was evidence of heterogeneity between the study estimates (*I*^*2*^ = 40%; heterogeneity, *P* = 0.09). Publication bias was not significant (Begg’s Test: *P* = 1.000; Egger’s Test: *P* = 0.639). Figure 2 expressed funnel plot of the incidence of pain relief comparing nalbuphine with morphine.

We consider probability distribution and checking the presence of scaling laws, using Bayesian methods (Bayesian meta-analyses). Instead of producing confidence intervals, Bayesian analyses produce credible intervals (sometimes called probability intervals). A 95% credible interval from a Bayesian analysis is a summary of the posterior distribution, such that the probability is equal to 95% that the true quantity is within the interval. This is a particularly intuitive way to express uncertainty, and is one of the most appealing aspects of a Bayesian analysis. WinBUGS software is now available for performing Bayesian analyses. Relative Risk of pain relief comparing nalbuphine and morphine using random effects model was 1.102(95% credible interval: 0.6697–1.627) based on 1000 simulated values by WinBUGS, which was similar with the outcome of conventional Meta-Analysis (RR: 1.01; 95% CI, 0.91 to 1.11). Thus our study was credible and stable with statistical methods.

### Side-effects

**Figure 3:**
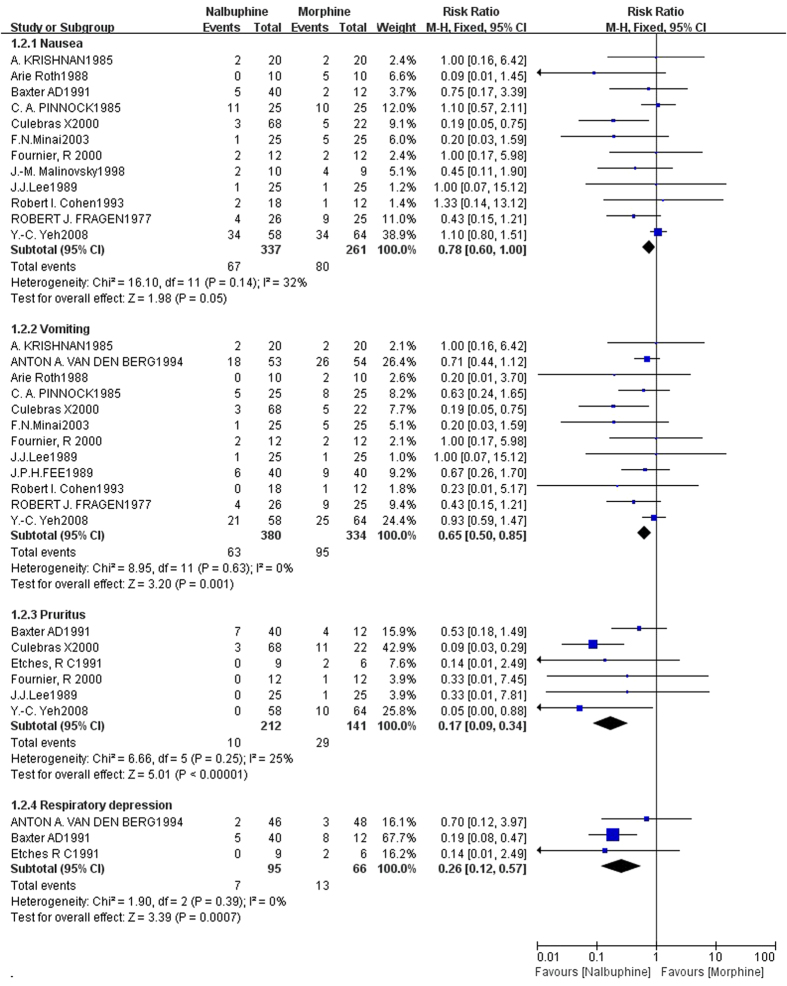
Forest plot of the incidence of nausea, vomiting, pruritus and respiratory depression comparing nalbuphine and morphine.

The pooled RRs comparing nalbuphine with morphine were 0.78(95%CI, 0.602–0.997; *P* = 0.048) for nausea, 0.65(95%CI, 0.50–0.85; *P* = 0.001) for vomiting, 0.17(95%CI, 0.09–0.34; *P* < 0.000) for pruritus,and 0.27(95%CI, 0.12–0.57; *P* = 0.001) for respiratory depression (Fig. 3, Table 2). The heterogeneity between the study estimates was not significant (pruritus *:I*^*2*^ = 25%, *P* = 0.63; nausea: *I*^*2*^ = 32%, *P* *=* 0.14; vomiting: *I*^*2*^ = 0.0%, *P* = 0.63; respiratory depression: *I*^*2*^ = 2%, *P* = 0.36). The results drawn from analyses suggested an advantage of nalbuphine over morphine regarding pruritus, nausea, vomiting and respiratory depression. Overall, from Table 2 we can see that the incidences of all the adverse events of nalbuphine and morphine, and the respective incidences of nausea, vomiting, pruritus and respiratory depression were 0.199, 0.16, 0.047 and 0.075 for nalbuphine, and 0.307, 0.284, 0.206 and 0.197 for morphine.

### Sensitivity Analysis and Meta Regression Analysis

To evaluate the influence of each study, sensitivity analysis was performed. On the one hand, when evaluating effect, a series of pooled RRs with 95% CIs produced similarly before and after eliminating each study at a time, suggesting that our results were robust and conservative.(Table 3) We can see that the largest portion of variance was explained when the study of Minai *et al.*^9^ was removed, I-squared decreased from 40% to 21% (*R*^*2*^ *=* 30%), and the value of *Tau-squared* was 0.00, which indicated that the goodness of fit of the model was good.

We conducted meta regression analysis in order to explore the source of heterogeneity in these respects of publication year, country, route of drug, disease of patients, study samplesize, and the Jadad score. First, we tested the influence of only one single attribute to the model (Table 1), but found no parameters was statistically significant. Second, we discussed the factor interactions and brought these covariates into models. None of these factors could have related to estimations of effect indeed.

While, stratified analysis was conducted by route, the estimates for the pooled RRs were 1.01(95%CI:0.89–1.16, *I*^*2*^ *=* 60%, *Tau*^*2*^ = 0.01) by intravenously, 1.09(95%CI:0.85–1.41, *I*^*2*^ *=* 10.0%, *Tau*^*2*^ = 0.00) by intramuscularly, and 1.03(95%CI: 0.70–1.51, *I*^*2*^ *=* 12%, *Tau*^*2*^ *=* 0.00) by intrathecally. (Table 2) We still found no significant difference between the two groups after the stratified analysis, and the route of administration may not cause heterogeneity. It is well recognized that efficacy of an analgesic is dependent upon the invasiveness of the surgical procedure as well as on the route of administration. Epidural/intrathecal administration is significantly more efficacious that intravenous or intramuscular administration. Therefore stratified analysis was conducted again, the pooled RRs were 0.99(95%CI: 0.94–1.04, *I*^*2*^ *=* 49%, *Tau*^*2*^ *=* 0.01) by intravenously and intramuscularly, and 0.96(95%CI: 0.76–1.21, *I*^*2*^ *=* 12%, *Tau*^*2*^ = 0.01) by intrathecally.

On the other hand, when evaluating safety, we could make comparisons with side-effects between the two opioids in different drug route. In intravenous and intramuscular administration, we found that Pooled risk ratios (RRs) for the incidence of adverse effects of nalbuphine versus morphine were 0.12(95%CI: 0.02–0.97) for Pruritus, 0.83(95%CI: 0.64–1.09) for Nausea, 0.72(95%CI: 0.55–0.94) for Vomiting. In intrathecal and epidural administration, we can see that pooled risk ratios (RRs) for the incidence of adverse effects of nalbuphine versus morphine were 0.22(95%CI: 0.07–0.66) for pruritus, 0.46(95%CI: 0.20–1.04) for nausea, 0.40(95%CI: 0.08–1.94) for vomiting. (Table 2)

## Methods

### Ethical consideration

This study was reviewed and approved by the ethical committee of School of Public Health, Tongji Medical College, Huazhong University of Science and Technology, Wuhan, China on 25 September 2014. The methods were carried out in accordance with the approved guidelines.

### Search Strategy

We searched the Cochrane Library and PubMed databases using Cochrane’s search strategy, confining the search to studies published between their inception and October 2014. We confined the search for full reports of randomized controlled trials. There were no language restrictions. Search details for each database are (“nalbuphine”[MeSH Terms] OR “nalbuphine”[All Fields] OR “nubain”[All Fields]) AND (Randomized Controlled Trial[ptyp] AND “humans”[MeSH Terms]). We got 176 hits in the Cochrane Library, and 177 hits for PubMed. We also reviewed citations listed in retrieved articles to identify additional studies.

### Study Selection

RCTs were eligible for analysis if the following criteria was met: (1) all patients should be randomly divided into nalbuphine and morphine groups; (2) Studies should evaluate efficacy and safety of nalbuphine compared with morphine; RCTs were eligible if they included at least one group receiving nalbuphine and one group receiving morphine. (3) Studies should provide the value of odds ratio/relative risk and 95% confidence interval; otherwise, data could be converted into relative risk and 95% confidence interval. Two reviewers independently searched literature with the same retrieval strategy, assessed retrieved titles and abstracts, and downloaded potentially relevant articles for further assessment.

### Data Extraction

Both investigators independently extracted the following information from the original articles: publication year, patient population details, patient type, interventions, number of cases and controls, number of patients that needed additional analgesic or patients with inadequate analgesia, number of patients with pain relief, the relative risk and 95% confidence interval, and the incidence of adverse effects. Disagreements on data extraction were resolved through discussion. While, our evaluation standard was as follows: (1) pain could be measured by the 100-mm visual analog scale (VAS), the 0–10 verbal rating score (VRS), the verbal category scale, or a four point score (0 = pain free; 1 = mild pain; 2 = moderate pain; 3 = severe pain), but it was patients whether needed additional analgesic during the early/late observation time after interventions that should be the basis of curative effect in our study evaluation; (2) respiratory depression was defined as a respiratory rate of <8 breaths/min or an arterial *PaCO*_*2*_ > 50 mmHg at any time postoperatively.

### Quality assessment

Study quality was judged by the Jadad scale score (5 points) according to the criteria proposed by Jadad and colleagues^21^, which evaluates studies based on randomization, blinding and dropouts. A study with a Jadad score between 3 and 5 was considered a high quality study^22^.

### Statistical Analysis

Data were extracted and summarized using relative risks with 95% confidence intervals (CIs) by the Review Manager 5.2. If the 95% CI included a value of 1 or *P* > 0.05, it was assumed that there was no statistically significant difference between nalbuphine and control^11^. We assessed heterogeneity of the study during this meta-analysis with chi-squared test and by calculating the value of I-squared, and *P* < 0.1 was considered statistically significant. Generally, if *I*^*2*^ > 56%, it prompts a significant heterogeneity, and trials were pooled using random effects model; if *I*^*2*^ < 31%, it indicates an insignificant heterogeneity, and trials were pooled using fixed effects model. Potential publication bias was assessed by Begg’s test^23^ and Egg’s test^24^. Sensitivity analyse and subgroup analysis could be conducted. Meta regression analysis by the stata statistical software version 10.0 was used to analyze sources of heterogeneity. *Tau-squared* expresses remel estimate of between-study variance, and the smaller the value is, the better the goodness of fit of the model becomes. *R*-*squared* represents how much the covariate currently into the model can explain the amount of variation between the research. All *P* values reported are two-sided.

## Discussion

Overall, 820 patients were included in the meta-analysis. This meta-analysis of randomized controlled trials provides the solid evidence to date regarding the efficacy and safety comparing nalbuphine with morphine. We discovered nalbuphine was comparable to morphine regarding analgesic efficacy. As we know, the evaluation of incidence of pain relief we extracted was not the most direct evidence, which was weakly expressed in clinical evidence for clinical effects, and thus, could not do better than the direct evidence of pain scores for evaluation. Even so, the outcomes we provided were worth considering. In addition, study quality of included studies had been considered, and in general, none of the ten studies^7^,^8^,^9^,^10^,^11^,^12^,^13^,^14^,^15^,^16^, which were made use of analyzing pain relief, was of low quality. There were four RCTs that got 3 scores, four RCTs that got 4 scores, and two RCTs that got 5 scores. In addition to this, allocation concealment had been evaluated, 5 of 10 studies reported allocation concealment^7^,^8^,^9^,^10^,^15^, which thus could present a more comprehensive evaluation of the possible bias in a randomized controlled study. All studies eligible for analysis used a randomized controlled design, which improved the reliability of the evidence.

The study by Etches *et al.*^20^ had a small sample size (morphine 5 mg, nalbuphine 10 mg, nalbuphine 20 mg: 6, 4, 5). In this study, 4 patients who received epidural nalbuphine 10 mg and all 5 who received epidural nalbuphine 20 mg got inadequate analgesia, and all 6 patients who received morphine had satisfactory analgesia (morphine vs. nalbuphine 10 mg, not significant; morphine vs. nalbuphine 20 mg, *P* *<* 0.01 )^20^. If the study by Etches *et al.*^20^ was included into evaluation of analgesic effects, the incidence of pain relief in pooled analyses was still no significant difference (RRs,1.00; 95% [CI], 0.89 to 1.12; *P* = 0.95; *I* ^*2*^= 51%). The study by Baxter *et al.*^25^ compared the analgesic efficacy and side-effects of epidural nalbuphine with epidural morphine in a randomised double-blind study in post-thoracotomy patients, revealing the pain scores were lowest in the morphine group (*P* *<* 0.01), which indicated an advantage in analgesic efficacy for morphine. Figure 1 shows the outcome of analgesic effects comparing nalbuphine with morphine, then we can find that the study by Minai *et al.*^9^ presented a positive result, which was one of factors causing heterogeneity. However, we can not ignore this research, for that its quality was all right.

Except for pain relief, there are many aspects to evaluate the effect of drugs, such as speed of drug action, efficacy of maintaining time, and pain scores. Culebras *et al.*^15^ conducted a study to compare the analgesic efficacy and adverse effects of intrathecal nalbuphine and intrathecal morphine for postoperative pain relief after cesarean deliveries. And it showed postoperative analgesia lasted significantly longer in the morphine group, compared with the nalbuphine groups (*P* *<* 0.0001).

As we know, pruritus is the most frequent side effect associated with spinal morphine^26^ that limits its use. This adverse effect is often difficult to treat and patients responds poorly to conventional treatments^27^. And The study by Somrat *et al.* reported that 3 mg of nalbuphine is effective in the treatment of intrathecal morphine-induced pruritus after cesarean delivery^28^. This study demonstrated a 20.6% incidence of morphine-induced pruritus by different kinds of routes. While, another two retrieved studies^15^,^16^, in which drugs were given by intrathecally, demonstrated a 38% incidence of intrathecal morphine-induced pruritus, and this is consistent with previously reported values^29^,^30^,^31^,^32^. Mixed agonist-antagonist opioid effects of nalbuphine have been reported for prevention of pruritus after epidural morphine^33^,^34^,^35^ Duration of action of intravenous nalbuphine is shorter than the duration of epidural morphine induced pruritus, and continuous intravenous infusion is needed to treat this side effect^34^.

Side effects such as pruritus, nausea, vomiting and urinary retention, are common^36^, but the most serious problem is respiratory depression^25^. Nalbuphine has a plateau effect on respiratory depression when given on its own^29^. It has been shown to reverse the respiratory depression from both intravenous^30^ and epidural^31^ opioids. The study by Baxter *et al.* reported that a 200 ug kg^−I^ bolus followed by a 50 ug kg^−I^ hr^−l^ infusion of nalbuphine may be administered to post-thoracotomy patients receiving epidural morphine, to prevent respiratory depression without causing significant side-effects or cardiovascular stimulation^25^.

When comparing nalbuphine with morphine, the pooled RRs were 0.17 for pruritus, and 0.27 for respiratory depression. The values of RRs were between 0.1 and 0.3, which indicated a strong correlation. Nalbuphine had a great advantage over morphine regarding these two side-effects of pruritus and respiratory depression. Our analysis also found other clinical advantages of nalbuphine, such as less nausea and vomiting. Sedation had been reported with postoperative analgesia with nalbuphine^31^,^32^,^37^, however, it was comparable to that produced by epidural morphine in the study by Baxter *et al.*^17^ There were only two retrieved studies^12^,^19^ that involved sedation, and the incidence of sedation were 0.114 with nalbuphine and 0.228 with morphine (Table 3).

Therefore, nalbuphine, which has a similar analgesia effect with morphine but has an advantage over morphine in some way, is another option for pain control.

In conclusion, our current meta-analysis indicates the analgesic efficacy of nalbuphine is comparable to morphine, but nalbuphine provides a better safety profile than morphine in the aspect of certain side-effects, especially related to pruritus and respiratory depression.

## Additional Information

**How to cite this article**: Zeng, Z. *et al.* A Comparision of Nalbuphine with Morphine for Analgesic Effects and Safety : Meta-Analysis of Randomized Controlled Trials. *Sci. Rep.* **5**, 10927; doi: 10.1038/srep10927 (2015).

## Figures and Tables

**Table 1:** Basic features and Meta Regression Analysis of the included studies for analgesic effects analysis.

**Reference**	**Factors**							
	**Year**	**Intervention (Nalbuphine/Morphine)**	**Country**	**Route**	**Disease**	**Observation Time**	**No. Of Patients**	**Jadad**
Robert J. FRAGEN	1977	0.1 Mg Kg^−I^ /0.1 mg Kg^−I^	North America	Intravenously	Gynaecology Related	Early	51(26/25)	4
Robert I. Cohen	1993	5 mg 0.5 ml^–I^/5 mg 0.5 ml^–I^	North America	Intravenously	Arthroscopic Surgery	Early	40(18/22)	4
F.N.Minai	2003	0.2 mg Kg^−I^/0.1 mg Kg^−I^	Asia	Intravenously	Gynaecology Related	Late	50(25/25)	4
Y.-C. Yeh	2008	1 mg Ml^–I^/1 mg Ml^−I^	Asia	Intravenously	Gynaecology Related	Late	122(58/64)	5
Anton A. Van Den Berg	1994	0.1-0.15 mg Kg^−I^/ 0.1-0.15 mg Kg^−I^	Asia	Intravenously	ENT Surgery	Late	107(53/54)	4
J.J.Lee	1989	0.4 mg Kg^−I^/ 0.2 mg Kg^−I^	North America	Intravenously	Burn Debridement Pain	Late	50(25/25)	3
J.P.H.Fee	1989	0.3 mg Kg^−I^/ 0.15 mg Kg^−I^	Europe	Intramuscularly	Hip Replacement	Late	80(40/40)	3
A. Krishnan	1985	0.3 mg Kg^−I^/ 0.2 mg Kg^−I^	Europe	Intramuscularly	Tonsillectomy	Late	40(20/20)	3
Culebras X	2000	0.2 mg/0.2 mg	Europe	Intrathecally	Gynaecology Related	Early	44(22/22)	5
Fournier, R	2000	400 μG/160 μG	Europe	Intrathecally	Hip Replacement	Late	24(12/12)	3
**Tau^2^**	0.01	--	0.01	0.01	0.00	0.00	0.01	0.01
***I***^***2***^	46%	--	46%	51%	40%	40%	46%	43%
***P***^a^	0.72	--	0.55	0.87	0.52	0.29	0.86	0.82

^a^*P* *<* 0.05 indicates the parameter or the factor is statistically significant in the meta regression.

**Table 2:** Pooled risk ratios (RRs) and 95% confidence intervals (CIs) for analgesic effects and safety comparing nalbuphine to morphine.

**Group**	**Events/Total**		**Heterogeneity of RRs**			**RR (95%CI)**
			***Chi*^*2*^**	P*	***I*^*2*^**	
	nalbuphine	morphine				
**Summary of Analgesic Effects**	215/299	217/309	0.01	0.09	40%	1.01(0.91–1.11)
***Stratified Analysis By Route***						
Intravenously	164/205	165/215	0.01	0.03	60%	1.01(0.89–1.16)
Intramuscularly	24/60	24/60	0.00	0.43	10%	1.09(0.85–1.41)
Intrathecally	27/34	28/34	0.00	0.29	12%	1.03(0.70–1.51)
**Summary of Safety**						
Pruritus	10/212	29/141	6.66	0.25	25%	0.17(0.09–0.34)
Nausea	67/337	80/261	16.10	0.14	32%	0.78(0.602–0.997)
Vomiting	63/380	95/334	8.95	0.63	0%	0.65(0.50–0.85)
Respiratory depression	7/93	13/66	1.90	0.39	0%	0.26(0.12–0.57)
***Stratified Analysis By Route*****Intravenously, Intramuscularly**#						
Pruritus	0/83	11/89	0.73	0.39	0%	0.12(0.02–0.97)
Nausea	57/217	71/215	10.53	0.23	24%	0.83(0.64–1.09)
Vomiting	58/300	88/227	5.14	0.82	0%	0.72(0.55–0.94)
**Intrathecally, By epidural**#						
Pruritus	10/129	18/52	5.08	0.17	41%	0.22(0.07–0.66)
Nausea	10/120	9/46	2.70	0.26	26%	0.46(0.20–1.04)
Vomiting&	5/80	7/34	2.06	0.15	51%	0.40(0.08–1.94)

^*^P values (two-sided) were based on the *Q* test of heterogeneity.

^&^When there was heterogeneity, the random effects model was used.

^#^stratified analysis was conducted, in which the routes by intravenously and intramuscularly were combined into one group, the routes by intrathecally and by epidural were combined into the other group.

**Table 3:** Outcomes produced after eliminating each study at a time.

**Study omitted**	**Heterogeneity of RRs**			**RR (95%CI)**	**P**
	**tau^2^**	**I-squared**	**P**		
Robert J. Fragen1977	0.01	38%	0.12	1.04(0.93–1.16)	0.567
Robert I. Cohen1993	0.01	40%	0.10	1.01(0.92–1.12)	0.79
F.N.Minai2003	0.00	21%	0.26	0.99(0.91–1.07)	0.75
Y.-C. Yeh2008	0.01	41%	0.09	1.03(0.90–1.18)	0.71
Anton A. Van Den Berg1994	0.01	29%	0.19	0.98(0.88–1.09)	0.69
J.J.Lee1989	0.01	43%	0.08	1.00(0.91–1.11)	0.99
J.P.H.Fee1989	0.01	45%	0.07	1.01(0.91-1.12)	0.84
A. Krishnan1985	0.01	43%	0.08	0.99(0.89–1.11)	0.92
Culebras X2000	0.01	46%	0.07	1.00(0.90–1.12)	0.96
Fournier, R 2000	0.01	42%	0.08	1.02(0.92–1.13)	0.71
